# Icatibant use in Brazilian patients with hereditary angioedema (HAE) type 1 or 2 and HAE with normal C1-INH levels: findings from the Icatibant Outcome Survey Registry Study^⋆^

**DOI:** 10.1016/j.abd.2021.09.009

**Published:** 2022-05-30

**Authors:** Anete S. Grumach, Marina T. Henriques, Maine L.D. Bardou, Daniele A. Pontarolli, Jaco Botha, Mariangela Correa, for the IOS Brazil Study Group

**Affiliations:** a Clinical Immunology, Faculdade de Medicina, Centro Universitario Saúde ABC, Santo André, SP, Brazil; b Takeda Pharmaceuticals International AG, Zurich, Switzerland; c Takeda Distribuidora Ltda, São Paulo, SP, Brazil

**Keywords:** Bradykinin, Bradykinin receptor, Brazil, Hereditary angioedema

## Abstract

**Background:**

Hereditary angioedema can be caused by C1-Inhibitor (C1-INH) deficiency and/or dysfunction (HAE-1/2) or can occur in patients with normal C1-INH (HAE nC1-INH).

**Methods:**

The Icatibant Outcome Survey (IOS; NCT01034969) registry monitors the safety and effectiveness of icatibant for treating acute angioedema.

**Objective:**

Present findings from Brazilian patients with HAE-1/2 and HAE nC1-INH participating in IOS.

**Results:**

42 patients were enrolled (HAE-1/2, n = 26; HAE nC1-INH, n = 16). Median age at symptom onset was significantly lower with HAE-1/2 vs. HAE nC1-INH (10.0 vs. 16.5y, respectively; p = 0.0105), whereas median age at diagnosis (31.1 vs. 40.9y; p = 0.1276) and the median time between symptom onset and diagnosis (15.0 vs. 23.8y; p = 0.6680) were numerically lower vs. HAE nC1-INH, respectively. One icatibant dose was used for > 95% of HAE attacks. Median (range) time-to-event outcomes were shorter for patients with HAE nC1-INH vs. HAE-1/2, including time to first administration (0.5 [0–96.0] vs. 1.0 [0–94.0]h, respectively), time from first administration to complete resolution (1.0 [0–88.0] vs. 5.5 [0–96.0]h, respectively), and total attack duration (7.0 [0.3–99.0] vs. 18.5 [0.1–100.0]h, respectively). Mean (SD) time from attack onset to resolution was significantly shorter for patients with HAE nC1-INH vs. HAE-1/2 (9.8 [18.7] vs. 19.6 [24.0]h, respectively; p = 0.0174). 83 adverse events (AEs) in 42 patients were reported; most were mild (66.3%) or moderate (13.3%) and non-serious (75.9%). The most common icatibant-related AE was injection site erythema (HAE-1/2, 34.6%; HAE nC1-INH, 18.8%).

**Study limitations:**

This was an observational study without a treatment comparator and that relied on patient recall.

**Conclusions:**

Findings demonstrate effectiveness and tolerability of icatibant in Brazilian HAE patients.

## Introduction

Hereditary angioedema (HAE) is a rare genetic disease associated with painful swelling episodes affecting subcutaneous and/or submucosal tissues. Attacks most often involve the head and neck, extremities, and abdomen. Laryngeal edema can be life-threatening due to the risk of suffocation.^1^, ^2^

HAE attacks recur with unpredictable frequency and severity, disrupting patients' personal and professional lives and imposing a heavy physical and emotional burden on patients, family members, and caregivers.^3^, ^4^, ^5^ Painful, potentially debilitating swelling episodes can negatively impact patients' work productivity, hamper the achievement of educational goals, and lead to anxiety about future attacks even during attack-free periods.^5^, ^6^ Per international HAE management guidelines, timely treatment of acute attacks and effective prevention of future attacks are integral aspects of care for patients with HAE.^7^, ^8^ The ultimate goal is to reduce attack frequency, duration, and severity, thus minimizing the burden of disease and helping to normalize patients' daily lives.^3^, ^8^

HAE type 1 (due to low levels and function of C1 inhibitor [C1-INH]) or type 2 (due to presence of dysfunctional C1-INH) is primarily caused by mutations in *SERPING1*, the gene encoding for C1-INH.^7^, ^9^ C1-INH is a serine protease that plays a pivotal role in downregulating activity in several signaling cascades, including the complement pathway, contact system (via the kallikrein-kinin cascade), coagulation pathway, and fibrinolysis.^10^ Within the kallikrein-kinin cascade, C1-INH is a pivotal inhibitor of factor XIIa and plasma kallikrein, which are key plasma proteases involved in the production of bradykinin, a potent vasodilator.^9^, ^10^ The absence of sufficient C1-INH function leads to uncontrolled activation of the kallikrein-kinin pathway and overproduction of bradykinin, the underlying cause of edema and swelling in patients with HAE type 1 or 2 (HAE-1/2).^9^, ^11^, ^12^

The presence of HAE in patients with normal C1-INH levels and function (HAE nC1-INH) is also increasingly being identified; pathogenetic pathways have not yet been fully elucidated. Several mutations in genes other than *SERPING1* have been reported to date (e.g., *FXII, kininogen-1 heavy chain, plasminogen, angiopoietin-1*), and recent findings suggest that the presence of a myoferlin gain-of-function variant may upregulate vascular endothelial growth factor-mediated signaling, leading to excessive vascular leakage. However, the underlying mutation often remains unknown.^12^, ^13^ As bradykinin is believed to play an important role in the pathogenesis of clinical manifestations of HAE nC1-INH,^11^, ^12^, ^14^ therapies that interfere with bradykinin production or bradykinin-mediated biological activities may, in principle, also be effective in patients with these forms of HAE.^11^ No controlled clinical trials in this setting have been conducted to date; however, evidence from case reports or observational studies show encouraging findings in some patients, including those treated with icatibant for acute HAE attacks.^15^, ^16^, ^17^ An urgent unmet need remains for further evaluation of treatment options in this patient population.

The Icatibant Outcome Survey (IOS; NCT01034969) is an ongoing, international, multicenter, observational, post-marketing registry study monitoring the safety and effectiveness of icatibant, a bradykinin B_2_ receptor antagonist, for the acute treatment of angioedema attacks. The IOS was initiated (by Shire, a Takeda company) in 2009 and ‒ as of September 30, 2019 ‒ has enrolled 1491 patients from 13 countries, including 10 European countries as well as Australia, Brazil, and Israel.

Formal characterization of HAE in Brazil began in 2006, with the creation of the Brazilian HAE registry by the Brazilian Network on the Diagnosis, Management and Treatment of HAE; knowledge of the Brazilian HAE patient population continues to grow.^18^ The estimated prevalence of HAE in Brazil is 4220 patients (based on an estimated 211 million people in Brazil in 2020 and an assumed HAE worldwide prevalence of ∼1:50,000).^7^, ^19^ Clinical features and genetic mutations in Brazilian patients with HAE are increasingly being reported, including findings in patients with HAE-1/2^20^, ^21^, ^22^ and in those with HAE nC1-INH.^16^, ^23^, ^24^, ^25^ Studies evaluating the quality of life in Brazilian patients with HAE demonstrate a heavy burden of disease in this population.^26^, ^27^ The majority of patients reported a continual fear of life-threatening attacks and demonstrated physical and emotional impairment; the negative impact was particularly noted with regard to vitality and social function.

The current analysis reports findings from Brazilian patients (recruited from one site in Brazil) who were enrolled in the IOS through September 30, 2019, including demographics, clinical characteristics, and treatment outcomes in patients with HAE-1/2 and those with HAE nC1-INH.

## Methods

### Patients and outcomes

The IOS is compliant with relevant global and local regulations and best practices and follows the Declaration of Helsinki principles and the International Conference on Harmonisation Good Clinical Practice Guidelines. Approval was granted by health authorities and local ethics committees. Written informed consent was provided by all patients (or their legally authorized representative).

**Figure 1:**
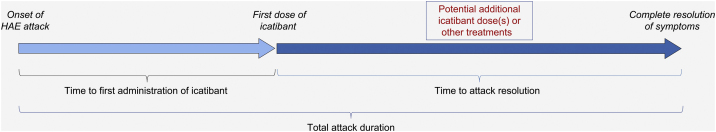
Treatment-related time-to-event effectiveness outcomes. Adapted from: Maurer M, et al.^28^. HAE, Hereditary Angioedema.

The IOS registry is open to all patients with HAE who have received at least one dose of icatibant. Of note, unlike in other IOS countries, patients in Brazil were required to be aged ≥18 years to participate (though icatibant is now approved for use in patients aged <18 years in Brazil). Per previously published descriptions of the IOS study design,^28^ data on patient demographics, clinical characteristics, and history of HAE attacks (including location, frequency, severity, duration, treatment outcomes, and tolerability) were collected at enrollment, and HAE attack-related information was recorded at regular follow-up visits (optimally every 6 months). Treatment-related time-to-event outcomes evaluated were time to first administration of icatibant (duration between onset of an attack and first icatibant administration); time to complete resolution (duration between first icatibant administration and complete resolution of all symptoms), and total attack duration (time between the onset of an attack and complete resolution of all symptoms) (Fig. 1).

Adverse events (AEs) were categorized per Medical Dictionary for Regulatory Activities System Organ Classification and Preferred Terms. AEs experienced prior to enrollment were recorded at IOS entry as part of patients' medical history, whereas AEs experienced after IOS enrollment were reported during follow-up visits. Although off-label use was documented in the IOS registry as part of the safety information to be reported according to regulatory standards, such events were not regarded as clinically meaningful and, as such, were analyzed separately from the current safety analysis of AEs.

### Statistical analysis

All statistical analyses were performed using SAS® software version 9.4 (SAS Institute, Cary, NC, United States). Quantitative outcome measures were reported as a number of observations, mean (Standard Deviation [SD]) or median, first quartile (25th percentile), third quartile (75th percentile), minimum, and maximum values. Statistical differences in baseline characteristics between patients with HAE-1/2 and patients with HAE nC1-INH were evaluated using the Wilcoxon test or Chi-Square test. Evaluations for time-to-treatment outcomes were performed using a mixed model for repeated measures on base ten log-transformed data. Severity comparisons (mild, moderate vs. severe, very severe) were performed using a generalized linear mixed model of repeated measures. Given the observational nature of this registry, all analyses were considered exploratory, and p-values were interpreted descriptively.

## Results

### Baseline characteristics

**Figure 2:**
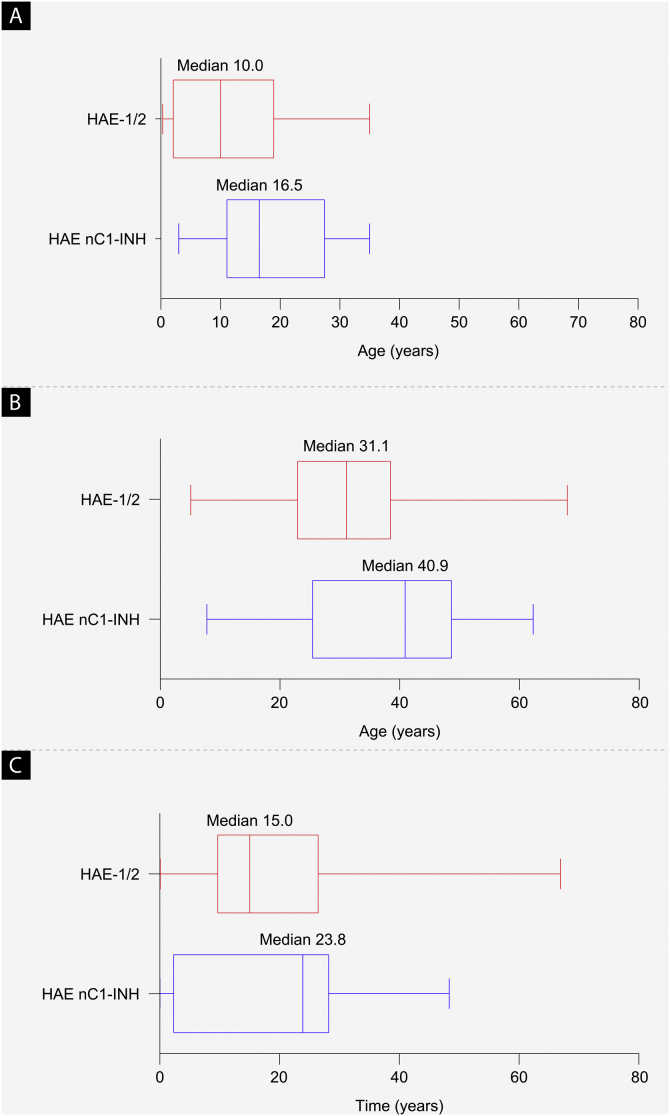
Baseline demographics. (a) Median age at hereditary angioedema symptom onset (p = 0.0105), (b) median age at diagnosis (p = 0.1276), and (c) median time from symptom onset to diagnosis (difference between age at first symptoms and age at diagnosis; p = 0.6680). The left and right edges of the boxes represent the 25th and 75th percentiles, respectively; box whiskers denote the minimum and maximum recorded values. HAE-1/2, Hereditary Angioedema with C1-inhibitor deficiency and/or dysfunction; HAE nC1-INH, Hereditary Angioedema with normal levels and function of C1-inhibitor.

As of September 30, 2019, a total of 42 patients from Brazil were enrolled in the IOS registry, representing 2.8% of the total patient enrollment (HAE-1/2, n = 26; HAE nC1-INH, n = 16). Demographic and clinical characteristics are shown in Table 1 and Fig. 2. The majority of patients were female (HAE-1/2, 84.6%; HAE nC1-INH, 100%). Most reported a positive family history (HAE-1/2, 80.8%; HAE nC1-INH, 93.8%). Median age at symptom onset was significantly lower in patients with HAE-1/2 than in those with HAE nC1-INH (10.0 vs. 16.5 years; p = 0.0105). Median age at diagnosis (31.1 vs. 40.9 years; p = 0.1276) and the median time between symptom onset and diagnosis (15.0 vs. 23.8 years; p = 0.6680) were numerically lower in patients with HAE-1/2 than in those with HAE nC1-INH, respectively, but differences were not statistically significant. At the time of data cutoff, information collected on the presence or absence of specific genetic mutations in patients with HAE nC1-INH was incomplete; findings from that analysis are thus not presented.

**Table 1:** Baseline characteristics.

Characteristic	HAE-1/2 (n = 26)	HAE nC1-INH (n = 16)	Overall (n = 42)
**Sex**			
n (MI)	26 (0)	16 (0)	42 (0)
Female, n (%)	22 (84.6)	16 (100.0)	38 (90.5)
Male, n (%)	4 (15.4)	0	4 (9.5)
**Age at HAE symptom onset (years)**			
n (MI)	23 (3)	16 (0)	39 (3)
Mean (SD)^a^	10.5 (9.4)	18.9 (10.1)	14.0 (10.4)
Median (range)	10.0 (0.3–35.0)	16.5 (3.0–35.0)	14.0 (0.3–35.0)
**Age at HAE diagnosis (years)**			
n (MI)	25 (1)	16 (0)	41 (1)
Mean (SD)^b^	31.6 (16.2)	37.6 (15.8)	33.9 (16.1)
Median (range)	31.1 (5.1–68.0)	40.9 (7.8–62.3)	33.8 (5.1–68.0)
**Time between HAE symptom onset and diagnosis (years)**			
n (MI)	22 (4)	16 (0)	38 (4)
Mean (SD)^c^	18.8 (17.8)	18.8 (15.9)	18.8 (16.8)
Median (range)	15.0 (0–66.9)	23.8 (0–48.3)	16.1 (0–66.9)
**Family history of HAE**			
n (MI)	24 (2)	16 (0)	40 (2)
Yes, n (%)^d^	21 (87.5)	15 (93.8)	36 (90.0)
No, n (%)^d^	3 (12.5)	1 (6.3)	4 (10.0)
**Age at IOS enrollment (years)**			
n (MI)	26 (0)	16 (0)	42 (0)
Mean (SD)	41.9 (13.0)	42.1 (13.8)	42.0 (13.2)
Median (range)	39.8 (19.0–0.7)	42.9 (18.3–64.3)	41.9 (18.3–70.7)

HAE, Hereditary Angioedema; HAE-1/2, Hereditary Angioedema with C1-inhibitor deficiency and/or dysfunction; HAE nC1-INH, Hereditary Angioedema with normal levels and function of C1-inhibitor; IOS, Icatibant Outcome Survey; MI, missing information; SD, Standard Deviation.

a p = 0.0105.

b p = 0.1276.

c p = 0.6880.

d Percentages exclude missing values.

### Number of attacks and number of icatibant administrations

A total of 165 icatibant-treated attacks were reported in 18 patients with HAE-1/2, and 63 icatibant-treated attacks were reported in 10 patients with HAE nC1-INH. One icatibant administration was used for the majority of attacks (95.7% in patients with HAE-1/2; 96.7% in those with HAE nC1-INH) (Table 2). It should be noted that concomitant rescue therapy with agents other than icatibant for the treatment of HAE attacks was used by some patients (Table 3). Concomitant rescue medications may have been used to help alleviate symptoms of, rather than treat, the attack. For example, analgesics and antiemetics may have been used to treat the pain, nausea, and vomiting that can accompany abdominal attacks, and fluids may have been administered as supportive care for the same attack. In some cases, patients used multiple rescue medications, but not necessarily for the same attack. The agents used for specific attacks were not recorded.

**Table 2:** Frequency of attacks and icatibant administrations.

	HAE-1/2	HAE nC1-INH
**Number of patients experiencing ≥1 icatibant-treated HAE attack**	18	10
**Number of attacks per patient**		
Mean (SD)	9.2 (9.7)	6.3 (4.5)
Median (Q1, Q3)	5.5 (1.0, 18.0)	6.0 (5.0, 8.0)
Range	1–29	1–17
**Percentage of attacks requiring 1, 2, or 4 icatibant administrations (%)**^a^		
1	95.7	96.7
2	4.3	1.7
4	0	1.7

HAE, Hereditary Angioedema; HAE-1/2, Hereditary Angioedema with C1-inhibitor deficiency and/or dysfunction; HAE nC1-INH, Hereditary Angioedema with normal levels and function of C1-inhibitor; Q, Quartile; SD, Standard Deviation.

a Based on a total number of 164 attacks (one missing) in patients with HAE-1/2 and a total of 60 attacks (three missing) in patients with HAE nC1-INH.

**Table 3:** Use of medications in addition to icatibant for treated HAE attacks during the follow-up period.

Medication, n (%)	HAE-1/2 (n = 164 [1 missing])	HAE nC1-INH (n = 63)	Total (n = 227)
Analgesics	7 (4.3)	5 (7.9)	12 (5.3)
Antiemetics	5 (3.0)	0	5 (2.2)
Antifibrinolytics	15 (9.1)	31 (49.2)	46 (20.3)
Antihistamines	3 (1.8)	1 (1.6)	4 (1.8)
Attenuated androgens	5 (3.0)	0	5 (2.2)
C1-INH concentrate	15 (9.1)	0	15 (6.6)
Fluids	1 (0.6)	0	1 (0.4)
Fresh frozen plasma	1 (0.6)	0	1 (0.4)
Other	5 (3.0)	1 (1.6)	6 (2.6)
Proton pump inhibitors	1 (0.6)	0	1 (0.4)
Spasmolytics	4 (2.4)	3 (4.8)	7 (3.1)

C1-INH, C1 Inhibitor; HAE, Hereditary Angioedema; HAE-1/2, Hereditary Angioedema with C1-inhibitor deficiency and/or dysfunction; HAE nC1-INH, Hereditary Angioedema with normal levels and function of C1-inhibitor.

Over the course of a mean (SD) 4.5 (1.66) years of follow-up (from IOS entry until date of data extract or discontinuation) for patients with HAE-1/2, and a mean (SD) 4.3 (1.42) years of follow-up for patients with HAE nC1-INH, the maximum number of attacks reported per patient was higher in patients with HAE-1/2 (29 attacks) than in those with HAE nC1-INH (17 attacks) (Table 2).

### Location and severity of attacks

**Figure 3:**
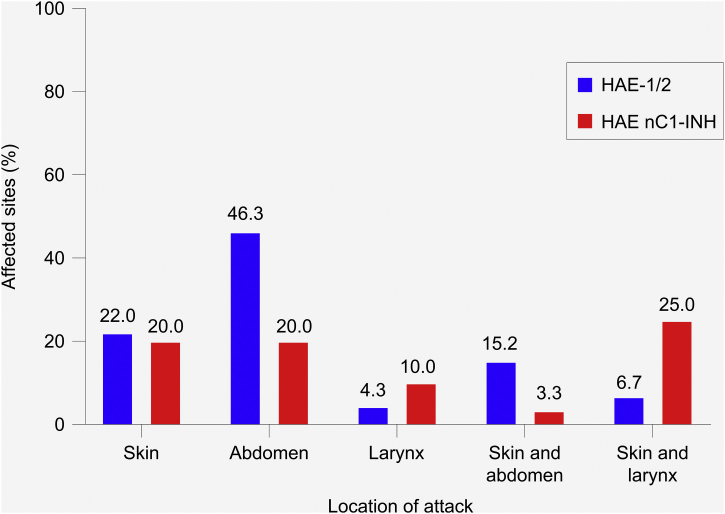
Most common location of hereditary angioedema attacks. HAE-1/2, n = 164 attacks (one missing); HAE nC1-INH, n = 60 attacks (three missing). Only the most common single sites and combinations of sites are shown; thus, percentages do not total 100%. Attacks affecting the skin refer to the following locations: arms, ears, eyelids, face, feet, genitals, hands, legs, lips, nose, torso, other, unknown. Abdominal attacks refer specifically to attacks affecting the gastrointestinal mucosa. HAE-1/2, Hereditary Angioedema with C1-inhibitor deficiency and/or dysfunction; HAE nC1-INH, Hereditary Angioedema with normal levels and function of C1-inhibitor.

In patients with HAE-1/2, most attacks (72.6%) affected a single site ‒ most commonly the abdomen (46.3%), followed by the skin (22.0%) (Fig. 3). The most common combination of sites involved the skin and abdomen (15.2%). In patients with HAE nC1-INH, attacks were equally likely to affect a single site or multiple sites (50% each). The skin and abdomen were the most common single sites affected (20% each). The most common combination of sites involved the skin and larynx (25.0%).

**Figure 4:**
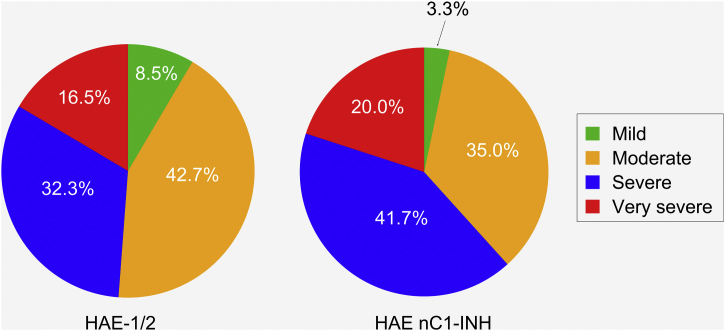
Severity of hereditary angioedema attacks prior to treatment. HAE-1/2, n = 164 attacks (one missing); HAE nC1-INH, n = 60 attacks (three missing). Attack severity was evaluated based on the following descriptions: Mild: mild interference with daily activities; Moderate: moderate interference with daily activities and no other countermeasures required; Severe: severe interference with daily activities and with or without other countermeasures; Very severe: very severe interference with daily activities and other countermeasures required. The difference between the two patient groups in the occurrence of mild/moderate vs. severe/very severe attacks was not significant (p = 0.1038). HAE-1/2, Hereditary Angioedema with C1-inhibitor deficiency and/or dysfunction; HAE nC1-INH, Hereditary Angioedema with normal levels and function of C1-inhibitor.

Patients with HAE nC1-INH were more likely than those with HAE-1/2 to experience severe or very severe attacks prior to treatment (61.7% vs. 48.8%, respectively). However, this difference (severe vs. very severe) was not significant (p = 0.8906), nor was the difference in the occurrence of mild/moderate vs. severe/very severe attacks (p = 0.1038) (Fig. 4).

### Treatment-related time-to-event outcomes

**Figure 5:**
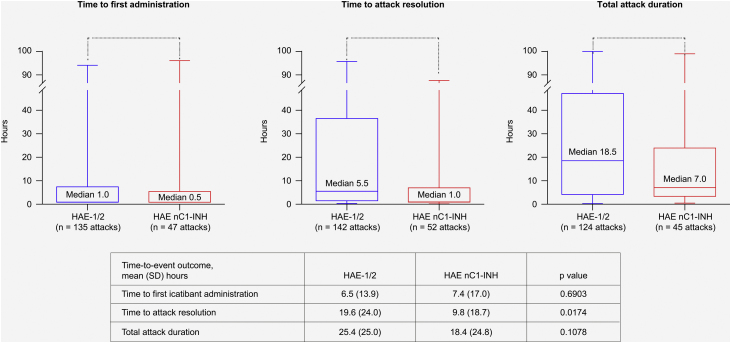
Time-to-event outcomes. The lower and upper edges of the boxes represent the 25th and 75th percentiles, respectively; box whiskers denote the minimum and maximum recorded values. HAE-1/2, Hereditary Angioedema type 1 or 2; HAE nC1-INH, Hereditary Angioedema with normal levels and function of C1-inhibitor; SD, Standard Deviation.

Treatment outcome data were available for up to 142 attacks in 17 patients with HAE-1/2, and for up to 52 attacks in eight patients with HAE nC1-INH. All median (range) time-to-event outcomes were shorter for patients with HAE nC1-INH (Fig. 5), including time from attack onset to first icatibant administration (HAE nC1-INH, n = 47 attacks, 0.5 [0–96.0] hours; HAE-1/2, n = 135 attacks, 1.0 [0–94.0] hours); time from first icatibant administration to complete symptom resolution (HAE nC1-INH, n = 52 attacks, 1.0 [0–88.0] hours; HAE-1/2, n = 142 attacks, 5.5 [0–96.0] hours); and total attack duration (HAE nC1-INH, n = 45 attacks, 7.0 [0.3–99.0] hours; HAE-1/2, n = 124 attacks, 18.5 [0.1–100.0] hours). Of note, the mean (SD) time from attack onset to resolution was significantly shorter for patients with HAE nC1-INH vs. HAE-1/2 (9.8 [18.7] hours vs. 19.6 [24.0] hours; p = 0.0174) (Fig. 5).

### Safety

A total of 83 events in 42 patients were reported, most of which were rated as mild (66.3%) or moderate (13.3%) in severity and non-serious (75.9%). None of the events were life-threatening or fatal. A total of 44 AEs in 16 patients were reported as possibly (four events) or probably (40 events) related to icatibant use. The most commonly occurring icatibant-related AE was injection site erythema, affecting 34.6% of patients with HAE-1/2 and 18.8% of those with HAE nC1-INH (Table 4). No unexpected events or new safety signals were detected.

**Table 4:** AEs considered as possibly or probably related to icatibant use (by Preferred Term)^a^.

AE, n (%)	HAE-1/2		HAE nC1-INH	
	Patients (n = 26)	Events (n = 58)	Patients (n = 16)	Events (n = 25)
**Injection site reaction**				
Erythema^b^	9 (34.6)	18 (31.0)	3 (18.8)	6 (24.0)
Application site pain	2 (7.7)	3 (5.2)	1 (6.3)	1 (4)
Bruise	‒	‒	1 (6.3)	1 (4)
Hemorrhage	1 (3.8)	1 (1.7)	‒	‒
**Hyperemia**	3 (11.5)	4 (6.9)	‒	‒
**Localized edema**	1 (3.8)	1 (1.7)	2 (12.5)	2 (8)
**Pain**	3 (11.5)	3 (5.2)	1 (6.3)	1 (4)
**Nausea**	1 (3.8)	1 (1.7)	‒	‒
**Edema**	1 (3.8)	1 (1.7)	‒	‒
**Angioedema**^c^	1 (3.8)	1 (1.7)	‒	‒

AE, Adverse Event; HAE-1/2, Hereditary Angioedema with C1-inhibitor deficiency and/or dysfunction; HAE nC1-INH, Hereditary Angioedema with normal levels and function of C1-inhibitor.

a Excluding reports of off-label use.

b Includes the Preferred Terms “Injection site erythema” and “Application site erythema”.

c This event was classified as a serious AE. The patient received two doses of icatibant prior to being hospitalized for 24 hours. In the hospital, the patient received fresh frozen plasma and was discharged the next day without sequelae.

## Discussion

Findings from the current analysis provide a valuable real-world representation of the use of icatibant in the treatment of patients with HAE-1/2 and HAE nC1-INH in Brazil. Though only one site in Brazil participated, a substantial number of patients were enrolled.

Baseline characteristics and clinical features of patients with HAE nC1-INH were generally consistent with previously published findings of French and Brazilian patient cohorts with HAE nC1-INH,^16^, ^23^, ^29^, ^30^ and findings in patients with HAE-1/2 were consistent with previously published characteristics in Brazilian patient cohorts with HAE-1/2.^18^, ^21^, ^22^ Compared with patients with HAE-1/2, patients with HAE nC1-INH had an older median age at symptom onset, older age at diagnosis, and a prolonged time between the onset of symptoms and diagnosis. Over the course of a similar follow-up period for the two patient groups, patients with HAE nC1-INH experienced fewer HAE attacks than those with HAE-1/2. This finding is consistent with a higher likelihood of disease-free intervals previously described in patients with HAE nC1-INH.^31^

In the present analysis, the location of attacks in patients with HAE-1/2 (most commonly affecting the abdomen or skin) were comparable with those previously reported in other Brazilian HAE-1/2 patient cohorts.^18^, ^22^ Patients with HAE nC1-INH were more likely to experience laryngeal attacks and less likely to experience abdominal attacks than patients with HAE-1/2, consistent with previous reports.^31^, ^32^ It should be noted, however, that several recently reported findings demonstrate the occurrence of abdominal attacks in > 50% of patients with HAE nC1-INH, especially in the presence of FXII mutations.^16^, ^23^, ^29^ The current findings further showed that regardless of HAE type, most attacks were effectively treated with one icatibant administration, as previously reported in French patients with HAE-1/2, as well as those with HAE nC1-INH enrolled in the IOS.^29^

The positive findings from this analysis of IOS patients from Brazil build upon the long-term effectiveness and safety of icatibant demonstrated from >10 years of experience with 1052 patients with HAE-1/2 in the IOS registry as of March 2019.^33^ In that analysis, based on data from 5253 treated attacks, complete resolution of attack-related symptoms occurred within a median of 6 hours after the first icatibant dose. The median total attack duration was 9 hours. Of 618 patients with HAE-1/2 exposed to icatibant during the follow-up period, 24 reported a total of 75 AEs that were considered possibly or probably related to icatibant use. The most frequent icatibant-related AEs were injection site erythema (29.3%) and asthenia (9.3%).

Non-controlled studies have shown encouraging outcomes with icatibant treatment in patients with HAE nC1-INH, including case reports and retrospective analyses.^15^, ^17^, ^30^, ^34^^,^^35^ Indeed, icatibant is among the treatment options recommended by current Canadian/International HAE treatment guidelines for the treatment of acute HAE attacks in patients with HAE nC1-INH (based on consensus level of evidence).^8^ Results from the current analysis of IOS Brazilian patients further emphasize the benefits of icatibant in patients with HAE nC1-INH. Findings showed a favorable trend for more rapid effects than in patients with HAE-1/2, including a shorter median time to complete resolution and shorter median total attack duration, though the only statistically significant difference between the two patient groups in this cohort was mean time from first icatibant administration to complete resolution of symptoms. Of note, the shorter median time from onset of attack to first icatibant administration for patients with HAE nC1-INH may have contributed to the improved time-to-event outcomes compared with patients with HAE-1/2, in line with previously published IOS data showing that earlier administration of icatibant results in more rapid resolution of HAE attacks.^28^

The safety profile of icatibant in the current analysis was comparable between patients with HAE-1/2 and those with HAE nC1-INH; injection site-related reactions were the most frequently reported AEs. Findings are consistent with the icatibant safety profile reported in pivotal controlled trials in patients with HAE-1/2^36^, ^37^ as well as in other post-marketing analyses of IOS patients and icatibant-treated patients with HAE nC1-INH in non-controlled studies.^29^, ^34^, ^38^

Various limitations are inherent in an observational study design, including a lack of treatment comparator and reliance on patient recall, potentially leading to underreporting of milder attacks or incomplete reporting of symptoms. Additionally, all analyses were post hoc, and the sample was not powered to show any differences; as such, results of comparisons between the two patient groups should be interpreted with caution. Despite these inherent limitations, however, registries such as the IOS are useful tools for gleaning insights about HAE beyond controlled clinical trial settings and for evaluating the effectiveness and safety of icatibant over extended time periods.

Of note, icatibant was launched in Brazil in 2009 and is approved by ANVISA (Brazil National Health Surveillance Agency), however, it has not been introduced in the official protocol from the Brazilian Ministry of Health. As such, government funding for its use is not currently provided, so patient access to this therapy remains a challenge. It is hoped that the continued reporting of icatibant effectiveness and safety findings, such as those reported herein, will help improve the accessibility of this agent for the treatment of HAE in Brazil.

## Conclusions

Characterization of various forms of HAE around the world continues to grow, paving the way to improved understanding of underlying genetic mutations, patient demographics, and treatment outcomes across varied HAE patient populations. The current analysis provides support for the effectiveness, safety, and tolerability of icatibant in Brazilian patients with HAE.

## Financial support

This research is sponsored by Shire Human Genetic Therapies, Inc., a Takeda company. Anete S. Grumach has a productivity scholarship CNPq. (309824/2021-4).

## Authors' contributions

Anete S. Grumach: Study conception and planning; data collection, analysis, and interpretation; preparation and writing of the manuscript and manuscript critical review; approval of the final version of the manuscript.

Marina T. Henriques: Data collection, analysis and/or interpretation; preparation and writing of the manuscript and manuscript critical review; approval of the final version of the manuscript.

Maine L.D. Bardou: Data collection, analysis and/or interpretation; preparation and writing of the manuscript and manuscript critical review; approval of the final version of the manuscript.

Daniele A. Pontarolli: Data collection, analysis and/or interpretation; preparation and writing of the manuscript and manuscript critical review; approval of the final version of the manuscript.

Jaco Botha: Data collection, analysis and/or interpretation; preparation and writing of the manuscript and manuscript critical review; approval of the final version of the manuscript.

Mariangela Correa: Data collection, analysis and/or interpretation; preparation and writing of the manuscript and manuscript critical review; approval of the final version of the manuscript.

## Conflicts of interest

Anete S. Grumach has been a speaker or consultant for BioCryst, Biotest, CSL Behring, and Takeda. Marina T. Henriques, Maine L. D. Bardou, and Daniele A. Pontarolli have no conflicts of interest to declare. J. Botha is an employee of and owns stock/stock options in Takeda. M. Correa was an employee of and held stock/stock options in Takeda at the time of this analysis.
